# Role of Resuscitation Promoting Factor-like Protein from *Nocardiopsis halophila*

**DOI:** 10.3390/microorganisms11020485

**Published:** 2023-02-15

**Authors:** Yufan Zhang, Jingjing Liu, Min Cao, Yujia Zhang, Xiumin Zhang

**Affiliations:** 1 College of Life Sciences, Hebei University, Baoding 071000, China; 2 Institute of Life Science and Green Development, Hebei University, Baoding 071000, China; 3 Key Laboratory of Microbial Diversity Research and Application of Hebei Province, Hebei University, Baoding 071000, China; 4 Engineering Laboratory of Microbial Breeding and Preservation of Hebei Province, Hebei University, Baoding 071000, China

**Keywords:** actinomycetes, extreme environment, *Nocardiopsis halophila*, *rpf* genes, resuscitation promoting factor (Rpf)

## Abstract

Resuscitation promoting factors (Rpf), a class of proteins secreted by gram-positive bacteria including actinobacteria, promote the resuscitation of dormant bacteria and spore germination. Here, we describe the reconstitution of the resuscitation promoting activity of the Rpf protein from *Nocardiopsis halophila* CGMCC 4.1195^T^ *in vitro* and *in vivo*. The Rpf protein was expressed in the host *Escherichia coli* BL21 codon plus (DE3) and was confirmed to have a significant resuscitation effect on the viable but non-culturable (VBNC) *N. halophila*. Subsequently, the *rpf* gene of *N. halophila* was knocked out. We found that the growth rate of the mutant strain (Δ*rpf*) was slower than that of the wild strain, and the former produced significantly shorter spores than the wild-type strain. Our results confirmed the activity of the Rpf protein in *N. halophila* to promote dormant bacteria resuscitation. This study will lay the foundation for the application of the Rpf protein from *N. halophila* to exploit actinomycetes resources.

## 1. Introduction

Rpf (resuscitation promoting factor) is a ≈16–17 kDa protein, first identified by Mukamolova et al. in 1998 in *Micrococcus luteus* [1], which could promote the resuscitation and growth of dormant, non-growing *M. luteus* [2]. The protein was also found to stimulate the growth of several other high G+C gram-positive bacteria [1]. Subsequently, the appearance of many sequenced genomes of various bacteria made clear that the Rpf of *M. luteus* was a member of a large family of the secreted bacterial Rpf-like proteins among the phylum Actinobacteria, including the representatives of *Mycobacterium*, *Corynebacterium*, *Nocardia*, *Rhodococcus* and *Streptomyces* genera. The number of the *rpf* gene orthologs for each species varies from 1 to 5 [3,4,5], and these collectively stimulate the growth of dormant bacteria [6,7,8,9,10]. To date, the Rpf-like proteins from *M. luteus*, *Mycobacterium tuberculosis*, *Mycobacterium smegmatis* and *Mycobacterium bovis*, have been used in vaccine development [6,11,12,13,14], diagnostic detection [15,16,17] and sewage treatment [18,19,20,21,22,23,24,25,26,27] due to its function of resuscitating dormant bacteria. In addition, our previous study confirmed that the Rpf protein of *M. luteus* could promote the isolation and culture of actinomycetes [28].

Actinomycetes are microorganisms that have considerable importance in the production of antibiotics [29]; they are responsible for producing more than 80% of antibiotics [30]. However, it has become increasingly difficult to isolate new actinomycetes and bioactive metabolites in normal soil [31]. During the past 20 years, a number of new actinomycetes strains which can produce bioactive compounds have been isolated from extreme or specific niches, including ocean, desert, and saline ecosystems [32,33,34,35], but these actinomycetes resources have not been fully exploited and utilized. The reason is that in these extreme environments, under certain as-yet-undefined stress conditions, actinomycetes tend to produce dormant exospores with thick cell walls to withstand adverse factors, resulting in difficulty germinating and growing under laboratory conditions. For dormant actinomycete bacteria, a return to active growth requires the so-called “resuscitation promoting factor” (Rpf) enzymes to break the thick protective cell wall [5,6,36,37,38,39,40,41].

Rpfs, acting as cell-wall-Lytic enzymes, have been implicated in the cleavage of dormant bacterial cell walls and the subsequent promotion of growth and metabolic reactivation in actinobacteria [42], which makes it possible to cultivate extreme environmental actinomycetes under laboratory conditions. *Nocardiopsis halophila*, a halophilic actinomycete isolated from a saline soil sample in Iraq [43], is an extreme environmental actinomycetes species. Our laboratory has previously analyzed the genome of *N. halophila* CGMCC 4.1195^T^, and found a single copy *rpf* gene with a size of 1251 bp encoding a protein of 416 amino acids. In this study, we aimed to clone and express the Rpf protein from *N. halophila* CGMCC 4.1195^T^ and verify its resuscitation function, laying the foundation for mining extreme environmental actinomycetes resources.

## 2. Materials and Methods 

### 2.1. Bioinformatics Analysis of Rpf Gene of N. halophila CGMCC 4.1195^T^

Multiple sequence alignment was performed on the UniProt portal (https://www.uniprot.org/) (accessed on 29 January 2023). Prediction of protein domain was carried out using HMMER (https://www.ebi.ac.uk/Tools/hmmer/) (accessed on 29 January 2023). Homology modeling was performed using Swiss-Model (https://swissmodel.expasy.org/interactive) (accessed on 29 January 2023) [44]. Alignment of the tertiary structure of two Rpf proteins was carried out using PyMol Version 2.5.4 [45]. MEGA X Version 10.2.0 [46] was used to construct phylogenetic tree.

### 2.2. Strains and Plasmids

The test strains, recipient strains and vectors used in this study are shown in Table 1.

### 2.3. Strain Culture

*N. halophila* CGMCC 4.1195^T^ was cultured in Salt Tolerance Medium (STM) broth (10 g starch, 2 g (NH_4_)_2_SO_4_, 1 g K_2_HPO_4_, 1 g MgSO_4_·7H_2_O, 1g NaCl, 2 g CaCO_3_, add an additional 100 g NaCl and 1000 mL deionized H_2_O, pH 7.2) at 28 °C, 180 r/min for 3–5 d. The cultures were centrifuged at 10,000 rpm for 10 min at 4 °C, and the pellets were washed with sterile water and stored at −20 °C. 

### 2.4. Gene Cloning and Construction of Expression Vector 

The PCR primers in Table 2 were used to amplify the *rpf* gene from *N. halophila* CGMCC 4.1195^T^ genomic DNA which was extracted using Bacterial Genome Small Quantity Extraction Kit (Sangon Biotech, Shanghai, China), referencing manufacturer’s instructions. The amplification procedure were as follows: predenaturation at 95 °C for 5 min, denatured 15 s at 95 °C, annealing 15 s at 65 °C, extension at 72 °C for 30 s, 30 cycles. The resulting PCR products were purified using a MiniBEST agarose gel DNA extraction kit (TaKaRa, Beijing, China) and sequentially digested with *Eco*RI and *Xho*lI and ligated with pMD18-T vector (TaKaRa, Beijing, China), which had been digested with the *Eco*RI and *Xho*lI, using T4 DNA ligase (TaKaRa, Beijing, China). The resulting ligation mixture (pMD18-T:*rpf*) was used to transform *E. coli* DH5α chemically competent cells (Sangon Biotech, Shanghai, China) referencing the manufacturer’s instructions and then plated on LB medium containing ampicillin (100 μg mL^−1^). A MiniBEST plasmid purification kit (TaKaRa, Beijing, China) was used to extract plasmid from transformants, and the predicted sequences confirmed by sequencing (Sangon Biotech, Shanghai, China). 

The purified pMD18-T:*rpf* and pET 28a(+) plasmid was all digested using the *Eco*RI and *Xho*lI, and both digested *rpf* gene insert and vector were cut from agarose gels and purified using a MiniBEST agarose gel DNA extraction kit (TaKaRa) and sequentially ligated using T4 DNA ligase (TaKaRa). The resulting ligation mix (pET 28a(+):*rpf*) was used to transform chemically competent cells of *E. coli* BL21 codon plus (DE3)(Sangon Biotech) and then plated on LB medium containing kanamycin (50 μg mL^−1^). The pET 28a(+):*rpf* plasmid extraction and the *rpf* gene sequences confirmation were as above.

### 2.5. Expression and Purification of Rpf Protein

The positive clone of *E. coli* BL21 codon plus (DE3) colonies including pET 28a(+):*rpf* were inoculated in 10 mL cultures supplemented with kanamycin (as above) and chloramphenicol (25 μg mL^−1^) and grown at 37 °C overnight, and then the cultures were inoculated into 500 mL of LB medium containing kanamycin (as above) and chloramphenicol (as above) and grown at 37 °C until an optical density of 0.6 to 0.8 at 600 nm (OD_600_), followed by induction with 50 mM IPTG (isopropyl-b-D-thiogalactopyranoside). Cells containing pET 28a(+):*rpf* were grown at 37 °C for a further 3 h before harvesting by centrifugation (10,000 r/min, 4 °C, 10 min). The cell pellets were collected and resuspended in Native IMAC lysis buffer (300 mM KCl, 50 mM KH_2_PO_4_, 5 mM imidazole) containing lysozyme (1mg·mL^−1^). The mixture was then sonicated by Ultrasonic Process or inanicebath for 15 min with 5 s pulse and 5 s break, and centrifuged at 10,000 r/min for 20 min at 4 °C. Recombinant Rpf was purified with Ni^2+^ affinity chromatography Protein Purification Kit (Bio-Rad Laboratories Inc., Hercules, CA, USA) with reference to the manufacturer’s instructions, and analyzed using SDS-PAGE. The protein was filtered and sterilized using a sterile 0.22 µm sterile millipore polytetrafluoroethylene membrane and finally stored in a refrigerator at −20 °C. The concentration of the recombinant Rpf protein was determined by the Lowry method [50].

### 2.6. Induction of VBNC State

*N. halophila* CGMCC 4.1195^T^ was inoculated in STM broth and cultured oscillatively at 200 r/min, 28 °C for 3-5 days. The cultures were collected and centrifuged at 4 °C at 6000 r/min for 10 min, and the cell pellets were washed using sterile saline solution (repeated three times). Finally, the cell pellets were re-suspended in sterile saline solution and stored in a refrigerator at 4 °C for at least three months (one year in this experiment) [18]. In the meantime, small amounts of the suspension were taken at two-week intervals and coated on the STM plate and cultured at 28 °C. When the growth could not be detected, it was considered to have entered the VBNC state.

### 2.7. Validation of Rpf Protein on the Resuscitation of VBNC N. halophila

To investigate the resuscitating ability of purified recombinant Rpf protein, a sample of VBNC *N. halophila* was diluted 10-fold with STM broth before adding purified recombinant Rpf protein to a final concentration of 1, 0.1 and 0.01 mg/mL, respectively, and then incubated on a shaker at 28 °C. The group without recombinant Rpf protein was used as the control group. Each group was repeated 3 times. The staining of resuscitated bacteria was observed by Confocal laser scanning microscope (FV3000, Olympus, Tokyo, Japan) on days 3, 5 and 7, on which propidium iodide (PI, red fluorescence) and fluorescein diacetate (FDA, green fluorescence) were used as fluorescent stains [51]. In addition, the growth of VBNC state bacteria under different concentrations of Rpf protein in STM broth was observed and monitored for 3 days using an automatic growth curve analyzer (Bioscreen C MBR, Helsinki, Finland). Each group had 3 replicates.

### 2.8. Construction of Rpf Gene Knockout Vector

DNA genome of *N. halophila* CGMCC 4.1195^T^ was extracted as outlined above and used to amplify the homologous arms of *rpf* with the primers shown in Table 2. The left arm was amplified using the primers L-F/L-R, and inserted into the plasmid pJTU1278 vector between *Xba*I and *Bam*HI sites to construct the recombinant plasmid pJTU1278: left. Similarly, the right arm was amplified using the primers R-F/R-R (Table 2) and inserted into the plasmid pJTU1278: left between *Bam*HI and *Hind*III sites to generate the recombinant plasmid pJTU1278: left/right. All the plasmids constructed in this work were verified by sequencing (Sangon Biotech, Shanghai, China). The constructed knockout vector was transferred into *E. coli* ET12567 (pUZ8002) for later use.

### 2.9. Deletion of N. halophila CGMCC 4.1195^T^ Rpf Gene

*E. coli* ET12567 (pUZ8002) with knockout vector was inoculated in LB liquid medium containing chloramphenicol (25 μg mL^−1^) and ampicillin (100 μg mL^−1^), and cultured overnight at 37 °C. On the second day, a small amount of culture was transferred to a fresh 50 mL LB medium containing chloramphenicol (as above) and ampicillin (as above), and cultured until an optical density of 0.5 to 0.6 at 600 nm (OD_600_). The cultures were centrifuged at 10,000 r/min for 10 min at 4 °C and the cell pellets were collected, washed with fresh LB medium 2–3 times, and then suspended with equal volume LB medium to be used as conjugation transfer donor. 

Spores of *N. halophila* CGMCC 4.1195^T^ from fresh cultures grown on the slant of STM were gently scraped off and added into 5 mL sterile water, and the spores suspension was placed in a sterilized 100 mL triangular flask with several sterile glass beads. The flask was shaken thoroughly to disperse the spores, filtered with sterilized cotton, and washed using 0.9% NaCl saline. Finally, the spores were resuspended with 0.9% NaCl saline and divided into 100 μL portions in 1.5 mL Eppendorf tubes.

The spore suspension was put into a 50 °C water bath pot for heat shock treatment for 10 min, and then transferred to 37 °C water bath pot for 0.5–1 h culture (pre-germination). The pre-germinated spores were washed in LB medium three times, and suspended in 100 μL LB medium as recipients. It was mixed with donors of 10^4^~10^8^ CFU (the recipients/donors were mixed in gradient ratios of 10:1, 1:1, 1:10, 1:30, respectively) for 30 min. The mixtures were spread on STM plates without antibiotics, blow-dried, and incubated at 28 °C for 16~20 h. The surfaces of the plates were covered with l mL sterile water containing nalidixic acid (75 μg mL^−1^) and thiostrepton (37.5 μg mL^−1^), and the white zygotes were observed after being blow-dried and cultured at 28 °C for 3–5 days. Finally, the zygotes were verified by colony PCR and sequencing, and the zygote lacking the *rpf* gene was used as deletion mutant Δ*rpf* strain. 

### 2.10. Comparison of the Growth of Wild Type Strain and Δrpf Strain

The wild-type strain and Δ*rpf* strain were coated on STM agar plates, respectively. The growth of the strains were observed at days 3, 5, and 7. At the same time, the sporulation was observed with optical microscope and scanning electron microscopy. In addition, we observed and monitored the growth of wild-type and Δ*rpf* strain in STM broth for 7 days using an automatic growth curve analyzer (Bioscreen C MBR, Helsinki, Finland). The above experiments were repeated 3 times.

### 2.11. Statistical Analysis

The GraphPad Prism (Version 9.4.1) was used for statistical analysis. Mean ± standard deviation (SD) for each parameter was calculated using the data of all three replicates. The unpaired T test was used to analyze the significance level (*p* < 0.05).

## 3. Results

### 3.1. Function Domains of Rpf Protein Encoded by Rpf Gene of N. halophila CGMCC 4.1195^T^

**Figure 1:**
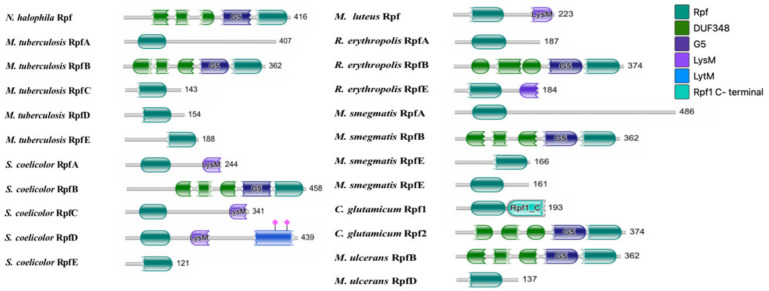
The function domain of Rpf proteins of *N. halophila*, *M. tuberculosis*, *S. coelicolor*, *M. luteus*, *R. erythropolis*, *M. smegmatis*, *C. glutamicum* and *M. ulcerans*.

Domains in the *N. halophila* CGMCC 4.1195^T^ *rpf* gene were predicted with HMMER (https://www.ebi.ac.uk/Tools/hmmer/) (accessed on 29 January 2023). The results showed that the Rpf coding chain has five domains as shown in Figure 1, comprising the following: three DUF348 domains (Pfam03990), which usually existed in the form of tandem repeats; one G5 domain, found in proteins involved in bacterial cell wall metabolism, which may have an adhesion function; and one Rpf catalytic domain, which acted to hydrolyze β-1,4 glycosidic bonds. Moreover, only the Rpf catalytic domains were ubiquitous in all Rpf-like proteins from *M. tuberculosis*, *M. smegmatis*, *Streptomyces coelicolor*, *Rhodococcus erythropolis*, *Corynebacterium glutamicum*, *Mycobacterium ulcerans* and *M. luteus*.

### 3.2. Homology of Rpf Proteins between N. halophila CGMCC 4.1195^T^ and of Other Species

**Figure 2:**
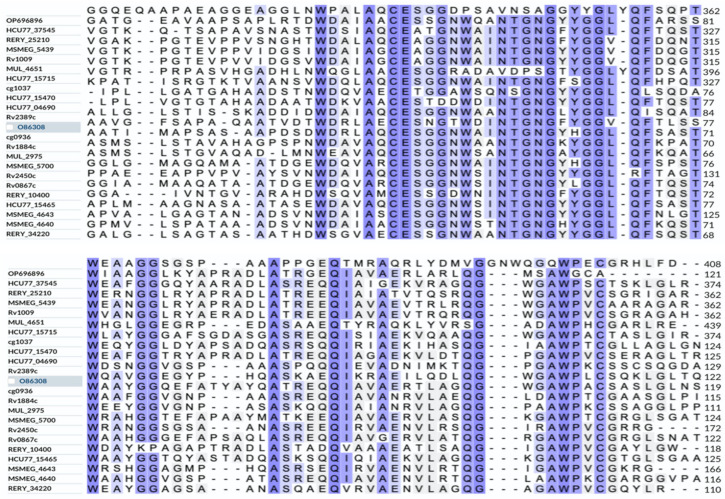
Multiple sequence alignment of Rpf (for resuscitation-promoting factors). Blue regions represent conserved domains. The alignment was performed on the UniProt portal (https://www.uniprot.org/align) (accessed on 29 January 2023). Sequence codes and sequences of *M. tuberculosis* are from UniProtKB database (https://www.uniprot.org/) (accessed on 29 January 2023) for the Rpfs of Rv2389c, Rv2450c, Rv1884c, Rv1009 and Rv0867c, *M. smegmatis* named MSMEG_5700, MSMEG_5439, MSMEG_4643 and MSMEG_4640, *M. luteus* named O86308, *S. coelicolor* named HCU77_15465, HCU77_15715, HCU77_15470, HCU77_04690 and HCU77_37545, *R.erythropolis* for RERY_10400, RERY_25210 and RERY_34220, *C. glutamicum* named cg0936 and cg1037, *M. ulcerans* named MUL_2975 and MUL_4651. Sequences of *N. halophila* are from NCBI Entrez database (http://www.ncbi.nlm.nih.gov/entrez) (accessed on 21 October 2022) the Rpf of OP696896.

Multiple sequence alignment was performed on the UniProt portal (https://www.uniprot.org/align) (accessed on 29 January 2023) for the amino acid sequences of the Rpfs of *N. halophila*, *M. luteus*, *S. coelicolor*, *M. tuberculosis*, *M. smegmatis*, *R. erythropolis*, *C. glutamicum* and *M. ulcerans*. It was found that they had in common a conserved domain with a similar amino acid sequence (approximately 75 amino acid residues) (Figure 2). This was consistent with the reports in the literature [3,6]. This conserved domain is also the catalytic domain of Rpf and possesses lysozyme activity, which can promote bacteria growth. The complete Rpf proteins have the same biological function as the individual Rpf conserved domain.

**Figure 3:**
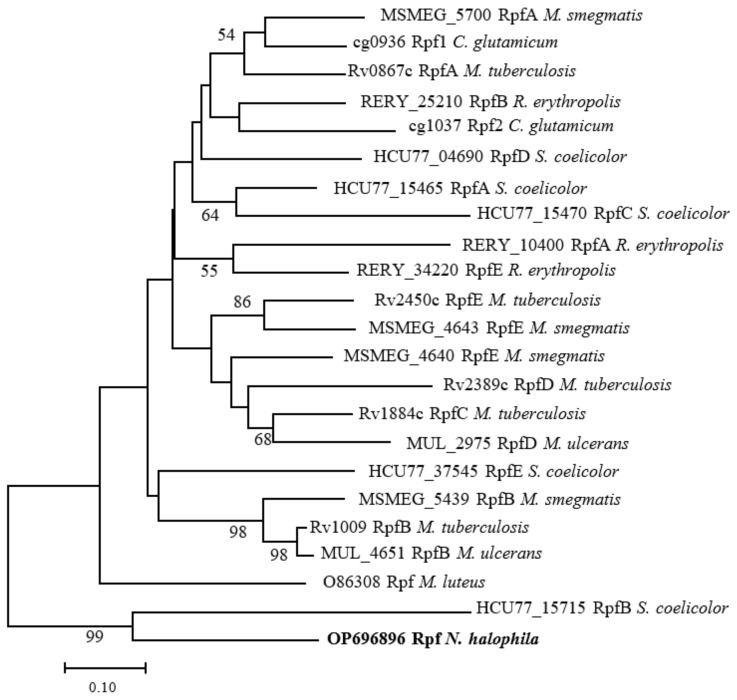
Phylogenetic tree based on amino acid sequences of Rpf proteins among *N. halophila*, *M. tuberculosis*, *M. smegmatis*, *M. luteus*, *S. coelicolor*, *R.erythropolis*, *C. glutamicum* and *M. ulcerans*. Phylogenetic tree was constructed using the maximum likelihood method in MEGA X Version 10.2.0. Numbers at nodes are based on 1000 resamplings; values above 50% are presented. Bar, 0.10 substitutions per amino acids position.

A phylogenetic tree was constructed using the maximum likelihood method in MEGA X Version 10.2.0 for the amino acid sequences of the Rpfs of *N. halophila*, *M. luteus*, *M. tuberculosis*, *M. smegmatis*, *R. erythropolis*, *S. coelicolor C. glutamicum* and *M. ulcerans*. The results showed that the Rpf protein of *N. halophila* and the Rpf B protein of *S. coelicolor* belong to the same branch of evolution (Figure 3), which proved that they were closely related.

### 3.3. Tertiary Structure Prediction of Rpf Protein 

**Figure 4:**
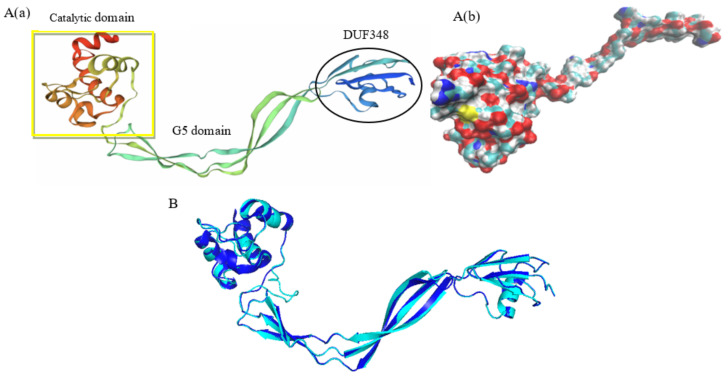
Tertiary structure of Rpf protein of *N. halophila* CGMCC 4.1195^T^. ((**A**)(**a**)): The structure shows a ribbon diagram; ((**A**)(**b**)): surface-filled model; (**B**): Alignment of the tertiary structure of two Rpf proteins. Grey represents Rpf protein of *N. halophila*; Blue represents the one of *M. tuberculosis*.

Swiss-Model was used to predict the tertiary structure of the Rpf protein of *N. halophila* CGMCC 4.1195^T^ (Figure 4A). The crystal structure of the Rpf protein included catalytic domain, G5 domain and DUF348, and the DUF348 domain adopted a compact fold, in which one α-helix was packed against a four-stranded β-sheet. The structure was consistent with the RpfB protein of *M*. *tuberculosis* [52]. 

Homology modeling of the Rpf protein of *N. halophila* CGMCC 4.1195^T^ was performed using Swiss-Model. The modeled Rpf protein of *N. halophila* and the template (5e27.1.A) of the RpfB protein of *M. tuberculosis* were the most closely correlated, and the sequence identity between them was 28.22%. The alignment of the tertiary structures of the two Rpf proteins was assessed using PyMol Version 2.5.4 [46]. The tertiary structures of the two proteins were well matched (Figure 4B). The calculation of the RMSD between the two proteins was carried out using SuperPose Version 1.0 online protein structure superposition software (http://wishart.biology.ualberta.ca/SuperPose/) (accessed on 29 January 2023). If all atoms are considered, the RMSD between the overall structure is 3.79 Å. If only the carbon skeleton is considered, the RMSD between the overall structure is 3.36 Å. From the RMSD value, we think that the two proteins are similar in structure.

### 3.4. Purification and Characterization of Rpf Protein from N. halophila CGMCC 4.1195^T^

The genomic DNA of *N. halophila* CGMCC 4.1195^T^ was used as a template to prepare *rpf* genes using primers in Table 2. The Rpf protein was expressed in *E. coli* BL21 codon plus (DE3) from plasmid pET 28a(+): *rpf*, and was purified from the cell extract with Ni^2+^ affinity chromatography. The purified recombinant Rpf protein was filtered and sterilized through a 0.22 μm sterile filter and adjusted to 2 mg/mL and stored in a refrigerator at −20 °C.

### 3.5. Validation of Rpf Protein on the Resuscitation of VBNC Bacteria 

**Figure 5:**
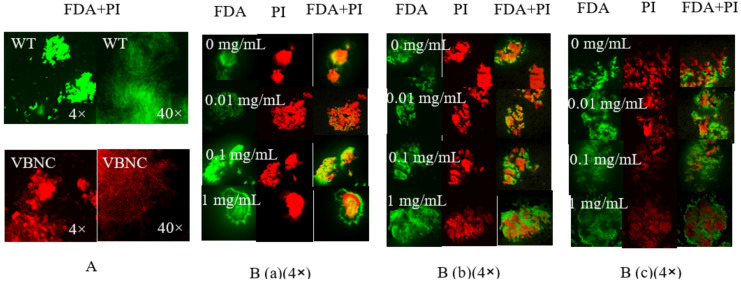
Growth of VBNC bacteria after the addition of Rpf from *N. halophila* CGMCC 4.1195^T^. (**A**). Wild-type strain (**a**) and VBNC strain (**b**). (**B**). Growth of VBNC bacteria after the addition of Rpf (0 mg/mL, 0.01 mg/mL, 0.1 mg/mL and 1 mg/mL) in 3 days (**a**), 5 days (**b**) and 7 days (**c**). In confocal fluorescence microscopy, dead bacteria were stained red by propidium iodide (PI) and live bacteria were stained green by fluorescein diacetate (FDA).

**Figure 6:**
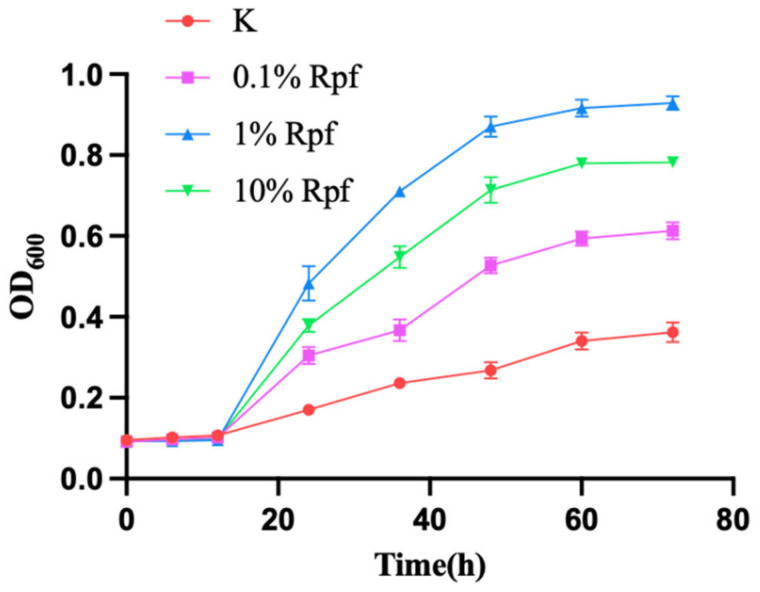
The growth curves of VBNC N. halophila CGMCC 4.1195^T^ at different Rpf protein concentrations.

The recombinant Rpf protein, at concentrations of 0.01, 0.1 and 1 mg/mL, respectively, was added to the bacteria solution that entered the VBNC *N. halophila* CGMCC 4.1195^T^. The resuscitating effect of the Rpf on the bacteria was observed using Confocal laser scanning microscope (FV3000, Olympus, Tokyo, Japan) on days three, five and seven and is shown in Figure 5. It was found that at day three there were significantly more living bacteria (green fluorescence) in the experimental group supplemented with the Rpf protein than in the control group without the Rpf protein (Figure 5), and the resuscitation effect on the VBNC *N. halophila* CGMCC 4.1195^T^ was most evident when 0.1 mg/mL of the recombinant Rpf protein was added to the medium. In addition, the same results were obtained from the growth curves of VBNC *N. halophila* CGMCC 4.1195^T^ at different Rpf protein concentrations (Figure 6). This also confirmed that the Rpf protein from *N. halophila* CGMCC 4.1195^T^ also had a resuscitation function similar to the *M. luteus* Rpf protein [2].

The Rpf protein obtained from *N. halophila* can be used for halophilic actinomycetes isolation from extreme halophilic environment samples in the future to further explore the effect of the Rpf protein on the culturability of halophilic actinomycetes. This will lay the foundation for further studies on the function of Rpf proteins derived from different actinomycetes.

### 3.6. Effects of Rpf Gene Deletion on Growth and Sporulation of Strain

**Figure 7:**
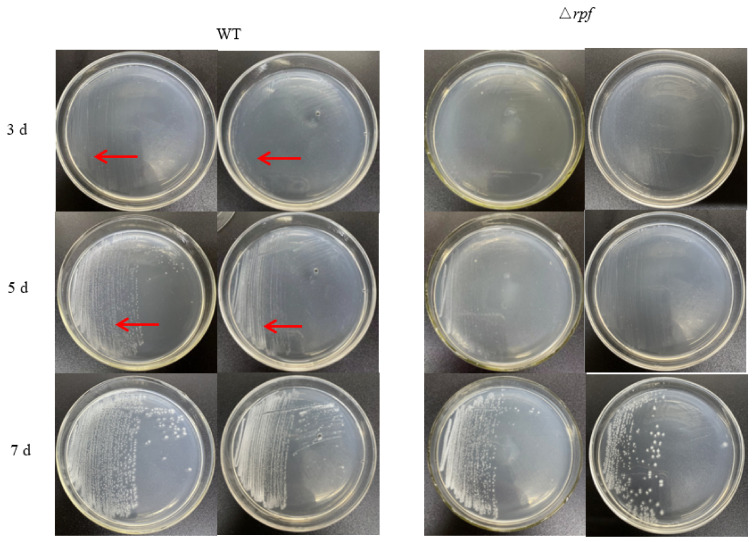
Growth of wild-type (WT) and *rpf* mutant strains (Δ*rpf*). On the third day, there were almost no colonies on the the mutant strain (Δ*rpf*) plate, while weak colonies appeared on the wild-type strain plate (Area indicated by the arrow); and on the fifth day, wild-type strain obviously formed a large number of white colonies (area indicated by the arrow), while the *rpf* mutant strain had less obvious and less white colonies.

**Figure 8:**
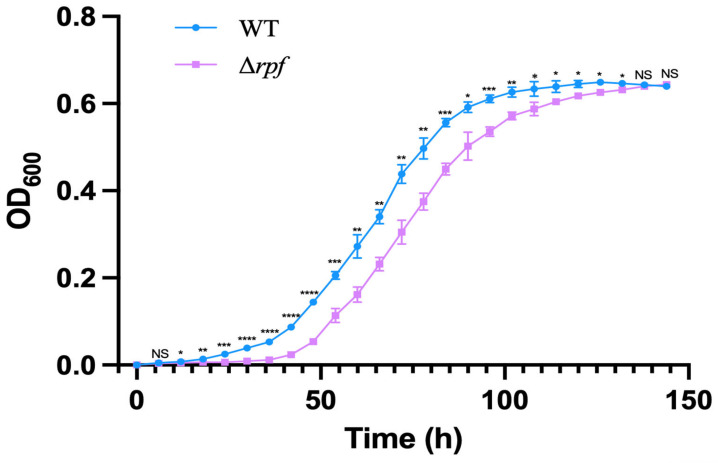
Growth curve of wild-type (WT) and rpf mutant strains (Δ*rpf*). The unpaired T test was used to analyze the significance level (*p* < 0.05), (*p* > 0.05, NS; *p* < 0.05, *; *p* < 0.01, **; *p* < 0.001, ***; *p* < 0.0001, ****) The values are plotted to represent the means ± standard deviations of three independent experiments.

**Figure 9:**
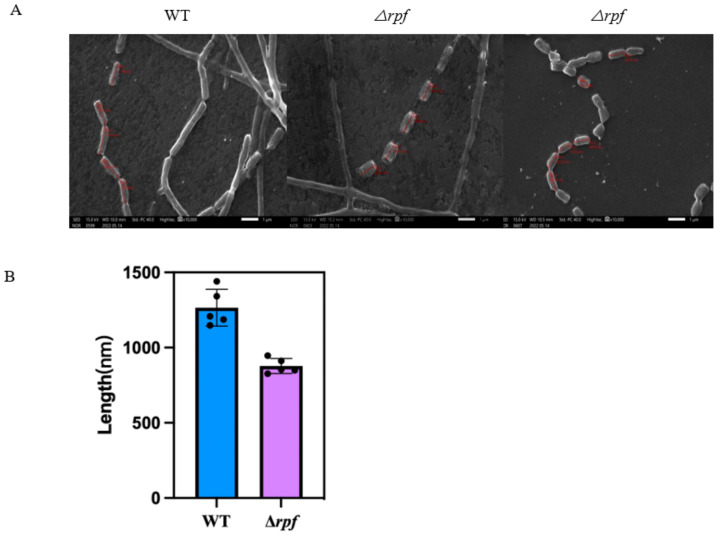
Effects of *rpf* gene deletion on sporulation. (**A**) Scanning electron micrographs of wild-type (WT) and *rpf* mutant strains (Δ*rpf*). Scale bar, 1 μm. (**B**) Analysis of spore length (*p* < 0.05). The unpaired T test was used to analyze the significance level (*p* < 0.05). The values are plotted to represent the mean ± standard deviations of at least five independent experiments for each strain.

In order to investigate the role of the *rpf* gene in *N. halophila* CGMCC 4.1195T, the growth of mutant and wild-type strains on STM plates was observed. The results showed that the growth rate of the mutant strain (Δ*rpf*) was slower than that of the wild strain in the early days. The wild-type strain had weak colonies at 3 days, however the Δ*rpf* bacteria could not be observed. At 5 days, the wild-type had more colonies than the Δ*rpf* group (Figure 7). Moreover, the growth curve also demonstrated that the mutant strain grew slightly slower than the wild-type strain (Figure 8), which is consistent with the results in Figure 7. Specifically, Combined with the growth curve, the growth of wild-type strain was significantly different from that of Δ*rpf* strain at 1–5 days (*p* < 0.05); at three days *p* = 0.0027, at five days *p* = 0.0007 (Figure 7). The same was true of vegetative growth in liquid culture (Figure 8). Moreover, we found that the mutant strain (Δ*rpf*) produced significantly shorter spores than the wild-type strain (*p* < 0.05) (Figure 9A,B). Whether this indicates that the deletion of *rpf* gene affects the spore morphology of the strain remains to be further verified.

## 4. Discussion

Rpf was first found in the culture supernatant of *M. luteus*, which could resuscitate VBNC bacteria [1]. Subsequently, the appearance of many sequenced genomes of various bacteria made clear that the Rpf of *M. luteus* was a member of a large family of the secreted bacterial Rpf-like proteins among many gram-positive (G+C)-rich bacteria belonging to the phylum Actinobacteria [3]. Moreover, the Rpf-like proteins derived from *M. tuberculosis* and *S. coelicolor* could also reactivate VBNC bacteria or dormant spores [9,36]. We previously found that there was a single copy of the *rpf* gene, with a size of 1251 bp, encoding an Rpf-like protein in the genome of *N. halophila*. Bioinformatics analyses showed that the Rpf-like protein had approximately 75 conserved amino acid residues similar to the *M. luteus* Rpf protein [42] (Figure 2), and the amino acid sequence of the Rpf-like protein was closely related to the RpfB protein of *S. coelicolo*r [6] (Figure 3). The results of a tertiary structure prediction for the Rpf protein showed that the modeled Rpf protein of *N. halophila* and the template (5e27.1.A) of the RpfB protein of *M. tuberculosis* were the most closely correlated, and the RMSDs between the two proteins were 3.79 Å (where all atoms were considered) and 3.36 Å (where only the carbon skeleton was considered), respectively. So, we speculate that the Rpf-like protein has a resuscitation function similar to the Rpf proteins of *M. tuberculosis* and *M. luteus*.

In order to verify the resuscitation effect of the Rpf-like protein of *N. halophila*, we cloned the *rpf* gene of *N. halophila* and expressed it heterologous in *E. coli* BL21 Codon Plus (DE3). Different concentrations of Rpf protein were added to VBNC bacteria and observed with confocal fluorescence microscopy. The results showed that the recombinant Rpf-like protein from *N. halophila* had a significant resuscitation effect on the VBNC *N. halophila*, and the resuscitation effect was most obvious when 0.1 mg/mL of the recombinant Rpf protein was added (Figure 5 and Figure 6), indicating that high concentrations of the Rpf protein inhibited the growth and recovery of VBNC *N. halophila*. This phenomenon is consistent with the results reported in the literature [53]. However, FDA and PI staining are not the best method because PI is not effective in distinguishing VBNC bacteria from bacterial death. Therefore, in future experiments we can try to adopt some other methods, such as the RT-PCR technique [54,55], immunological method [56], flow cytometry [57,58,59], nucleic acid dye, western blotting and so on.

In addition, in order to investigate the role of the *rpf* gene in *N. halophila* CGMCC 4.1195^T^, we constructed the *N. halophila* CGMCC 4.1195^T^ mutant strain with *rpf* gene deletion, and the growth of mutant and wild-type strains on STM plates was observed. The results showed that there were no significant differences in growth between the mutant strain and the wild-type strain (Figure 7 and Figure 8), but we found that the mutant strain sporulated slightly later than the wild-type strain, and had shorter spore sizes than the wild-type strain (Figure 9). Whether this indicates that the deletion of *rpf* gene can affect the spore morphology of the strain remains to be further verified.

It was reported that loss of the *rpf* gene was correlated with a delay in the onset of germination [6]. However, our experiment has not provided persuasive evidence, which we will need to complete in the future. In addition, bacterial spore germination is a complex physiological process. Usually, *Bacillus subtilis* forms heat- and desiccation-resistant spores under conditions of nutrient limitation [53]. These dormant spores germinate when stimulated by various nutrient sources. However, the molecular events leading to successful spore dormancy and later to germination remain to be fully elucidated [60]. The survival of an organism relies on its capacity to quickly respond and adapt to a constantly changing environment. Underlying this adaptive potential is the ability of cells to sense and transduce external and internal signals. Protein kinases, together with their cognate phosphatases, play a central role in signal transduction by catalyzing reversible protein phosphorylation. Eukaryote-like serine/threonine kinases and phosphatases are common in bacteria, such as such as PknB in *Mycobacterium tuberculosis* [54]; they might be able to sense and transduce external and internal signals and play an important role in spore germination. Moreover, Virmani et al. (2019) [61] reported that the spores of *Bacillus anthracis* inherit cellular components as phenotypic memory from the parent cell, and this memory plays a critical role in facilitating the spores’ revival. They found that increased expression of enolase (Eno) in the sporulating mother cell decreases germination efficiency. Eno is phosphorylated by the conserved Ser/Thr protein kinase PrkC which decreases the catalytic activity of Eno, and phosphorylation also regulates Eno expression and localization, thereby controlling the overall spore germination process [61]. There was evidence that spore germination was inhibited at the outgrowth step in the *rpf* null strain of *Streptomyces* [37]. However, whether Rpf protein is also regulated by Ser/Thr protein kinases to promote spore germination remains to be further investigated.

In short, based on the resuscitation function, the Rpf protein of *N. halophila* obtained in this study can be used to mine halophilic actinomycetes resources from extreme halophilic environment samples, and to improve the culturability of halophilic actinomycetes in the future. This will lay the foundation for further studying the function of Rpf proteins of different actinomycetes. Although this study did not focus on the relationship between the structure and function of Rpfs, nor the detailed mechanism of Rpf protein promoting spore germination, we plan to take them as the contents of further in-depth research in the future.

## Figures and Tables

**Table 1:** Strains and plasmids used in this study.

Strain or Plasmid	Description	Source or Reference
Strains		
*E. coli* DH5α	Strain used for cloning and plasmid maintenance	Sangon Biotech
*E. coli* BL21 codon plus (DE3)	Strain used for protein expression	Sangon Biotech
*E.coli* ET12567(pUZ8002)	*Dam dcm*, with transmobilizing plasmid pUZ8002	[47,48]
*N.halophila* CGMCC 4.1195^T^	Strain used for amplification of *rpf* gene.	[43]
Plasmids		
pMD18-T	Amp^r^ cloning vector	TaKaRa
pMD18-T:*rpf*	pMD18-T containing *rpf* from strain CGMCC 4.1195^T^	This study
pET 28a(+)	Amp^r^ expression vector	TaKaRa
pET 28a(+):*rpf*	pET 28a(+) containing *rpf* from strain CGMCC 4.1195^T^	This study
pJTU1278	Conjugation vectors used for *rpf* gene knockout	[49]
pJTU1278: left arm	pJTU1278 carrying the homologous arm of *rpf*	This study
pJTU1278: left/Right	pJTU1278 carrying the left and right homologous arms of *rpf*	This study

**Table 2:** Primers used in the present study.

Fragment	Primers	Sequence (5′-3′)	Restriction Enzyme
*rpf*	*rpf*-F	G**GAATTC**GGAAGAACCGTGCGCAAT	*Eco*RI
	*rpf*-R	CCG**CTCGAG**CATATGCTCAGTCGAACAGGTGGC	*Xho*lI
Left homologous arm of *rpf*	L-F	GC**TCTAGA**GGGCCTCAGGGGAGCTCCACGC	*Xba*I
	L-R	CG**GGATCC**GCCGCCGCGGCTCCGGTG	*Bam*HI
Right homologous arm of *rpf*	R-F	CG**GGATCC**GCCAGTGGCCCGAATGCG	*Bam*HI
	R-R	CCC**AAGCTT**GCCAGGAGACGGTGGCCC	*Hin*dIII

## Data Availability

All data and materials as well as software application or custom code support their published claims and comply with field standards.
